# Macrophage Migration Inhibitory Factor (MIF) Deficiency Exacerbates Aging-Induced Cardiac Remodeling and Dysfunction Despite Improved Inflammation: Role of Autophagy Regulation

**DOI:** 10.1038/srep22488

**Published:** 2016-03-04

**Authors:** Xihui Xu, Jiaojiao Pang, Yuguo Chen, Richard Bucala, Yingmei Zhang, Jun Ren

**Affiliations:** 1 Center for Cardiovascular Research and Alternative Medicine, University of Wyoming College of Health Sciences, Laramie, WY 82071 USA; 2 Department of Emergency, Qilu Hospital of Shandong University, Jinan, Shandong, 250012, PR China; 3 Department of Medicine, Yale School of Medicine, New Haven, CT 06520 USA; 4 Shanghai Institute of Cardiovascular Diseases, Zhongshan Hospital, Fudan University, Shanghai 200032, PR China

## Abstract

Aging leads to unfavorable geometric and functional sequelae in the heart. The proinflammatory cytokine macrophage migration inhibitory factor (MIF) plays a role in the maintenance of cardiac homeostasis under stress conditions although its impact in cardiac aging remains elusive. This study was designed to evaluate the role of MIF in aging-induced cardiac anomalies and the underlying mechanism involved. Cardiac geometry, contractile and intracellular Ca^2+^ properties were examined in young (3–4 mo) or old (24 mo) wild type and MIF knockout (MIF^−/−^) mice. Our data revealed that MIF knockout exacerbated aging-induced unfavorable structural and functional changes in the heart. The detrimental effect of MIF knockout was associated with accentuated loss in cardiac autophagy with aging. Aging promoted cardiac inflammation, the effect was attenuated by MIF knockout. Intriguingly, aging-induced unfavorable responses were reversed by treatment with the autophagy inducer rapamycin, with improved myocardial ATP availability in aged WT and MIF^−/−^ mice. Using an *in vitro* model of senescence, MIF knockdown exacerbated doxorubicin-induced premature senescence in H9C2 myoblasts, the effect was ablated by MIF replenishment. Our data indicated that MIF knockout exacerbates aging-induced cardiac remodeling and functional anomalies despite improved inflammation, probably through attenuating loss of autophagy and ATP availability in the heart.

Advanced aging, an irreversible biological process, is a major independent risk factor for cardiovascular in particular heart diseases^1^,^2^,^3^. Aging has been shown to promote unfavorable cardiac structural and functional alterations, including increased left ventricular wall thickness and chamber size, alterations in diastolic filling pattern such as prolonged diastole, interstitial fibrosis and decreased myocardial contractile capacity, all of which contribute to the ever-rising cardiac morbidity and mortality in the elderly^1^,^2^,^3^,^4^. Ample of clinical and experimental studies have suggested dramatically declined cardiac performance with aging^1^,^5^,^6^,^7^. Although several mechanisms have been postulated for the biology of aging over the last decades including oxidative stress and mitochondrial damage^3^,^8^,^9^, the precise mechanism underlying aging-induced cardiac anomalies remains elusive.

Autophagy is an evolutionarily conserved intracellular pathway for bulk degradation of aged or damaged proteins and organelles, generating free amino acids and fatty acids for ATP synthesis^10^,^11^. Basal autophagy has been considered to carry an essential permissive role in the control of cardiac homeostasis in physiological conditions^12^,^13^. Not surprisingly, autophagy is susceptible to a number of pathological insults, including obesity^14^,^15^, diabetes mellitus^16^,^17^, nutrition restriction^18^, ischemia/reperfusion^13^, pressure overload^19^,^20^ and aging^5^,^6^,^21^, leading to the derangement of cardiac autophagy, *en route* to compromised cardiac geometry and function. For example, dampened autophagy has been deemed to contribute to the pathogenesis of various aging-related diseases, in multiple organs including the heart^5^,^6^,^21^ and liver^22^,^23^. Extended longevity and improved cardiac performance have been confirmed in mouse aging models with induction of autophagy using the mammalian target of rapamycin (mTOR) inhibitor rapamycin^24^,^25^.

Macrophage migration inhibitory factor (MIF), originally identified as an inflammatory cytokine secreted from T-cells^26^,^27^, is ubiquitously expressed in various organs including hearts and livers^28^. MIF is believed to play an indispensable role in cardiac homeostasis under an array of pathophysiological conditions, including ischemia/reperfusion^28^, nutrition restriction^18^, high fat diet intake^15^, pressure overload^29^, and doxorubicin-induced cardiac anomalies^18^. Earlier findings from our group suggested impaired MIF-driven activation of AMPK in senescence, thus to contribute to the increased susceptibility to myocardial ischemic injury with aging^30^. However, the precise role of MIF in aging-induced changes in cardiac geometry and contractile function still remain elusive. To this end, this study was designed to evaluate the impact of MIF on aging-induced cardiac geometric and functional injuries, and the underlying mechanisms with a special focus on autophagy and inflammation, given the established autophagic and pro-inflammatory responses for MIF^15^,^18^,^28^,^29^. To examine the effect of MIF knockdown and replenishment on premature cell senescence, an established doxorubicin-induced model of premature senescence was employed in H9C2 myoblasts^31^,^32^,^33^. C_12_FDG, a fluorogenic substrate for β-galactosidase, was used to detect SA-β-gal as a marker for cellular senescence^22^.

## Materials and Methods

### Experimental animals

All animal procedures performed here were approved by the Animal Care and Use Committee at the University of Wyoming (Laramie, WY, USA) and were in compliance with the Guide for the Care and Use of Laboratory Animals published by the US National Institutes of Health (NIH Publication No. 85–23, revised 1996). In brief, young (3–4 month-old) and old (24 month-old) male MIF knockout (MIF^−/−^) mice and their C57BL/6 littermates [served as wild-type (WT)] were housed in a climate-controlled environment (22.8 ± 2.0 °C, 45–50% humidity) with a 12/12–light/dark cycle with access to food and water *ad libitum* until experimentation^22^.

### Intraperitoneal glucose tolerance test (IPGTT)

Mice were fasted for 12 hrs and were given an intraperitoneal (i.p.) injection of glucose (2 g/kg body weight). Blood samples were drawn from tail vein immediately before the glucose challenge, as well as 15, 60 and 120 min thereafter. Blood glucose levels were determined using an Accu-Chek glucose analyzer^34^.

### Administration of rapamycin

Rapamycin treatment was performed as described^29^,^35^. In brief, rapamycin (LC Laboratories) was dissolved in ethanol and resuspended in vehicle (0.25% PEG, 0.25% Tween-80) at a final concentration of 1 mg/ml. Rapamycin (i.p., 2 mg/kg/d) or vehicle was given male WT and MIF^−/−^ mice (22-month old) for 8 weeks. The dosage and duration of rapamycin treatment were chosen based on previous reports^25^,^29^,^35^.

### Echocardiographic assessment

Cardiac geometry and function were evaluated in anesthetized (ketamine 80 mg/kg and xylazine 12 mg/kg, i.p.) mice using the two-dimensional guided M-mode echocardiography (Philips SONOS 5500) equipped with a 15–6 MHz linear transducer (Phillips Medical Systems, Andover, MD, USA). The chests were shaved and mice were placed in a shallow left lateral position on a heating pad. Using the 2-dimensional (2-D) parasternal short-axis image obtained at a level close to papillary muscles as a guide, a 2-D guided M-mode trace crossing the anterior and posterior wall of LV was obtained at a sweep speed of 50 mm/sec. The echocardiographer was blind to the identity of mice examined. Caution was taken to avoid excessive pressure over the chest, which could induce bradycardia and deformation of the heart. Left ventricular (LV) anterior and posterior wall dimensions during diastole and systole were recorded from three consecutive cycles in M-mode using method adopted by the American Society of Echocardiography. Fractional shortening was calculated from LV end-diastolic (EDD) and end-systolic (ESD) diameters using the equation (EDD-ESD)/EDD*100^14^.

### Isolation of murine cardiomyocytes

Hearts were rapidly removed from anesthetized mice and mounted onto a temperature-controlled (37 °C) Langendorff system. After perfusion with a modified Tyrode’s solution (Ca^2+^ free) for 2 min, the heart was digested with a Ca^2+^-free KHB buffer containing liberase blendzyme 4 (Hoffmann-La Roche Inc., Indianapolis, IN, USA) for 20 min. The modified Tyrode solution (pH 7.4) contained the following (in mM): NaCl 135, KCl 4.0, MgCl_2_ 1.0, HEPES 10, NaH_2_PO_4_ 0.33, glucose 10, butanedione monoxime 10, and the solution was gassed with 5% CO_2_–95% O_2_. The digested heart was then removed from the cannula and left ventricle was cut into small pieces in the modified Tyrode’s solution. Tissue pieces were gently agitated and pellet of cells was resuspended. Extracellular Ca^2+^ was added incrementally back to 1.20 mM over 30 min. A yield of at least 60–70% viable rod-shaped cardiomyocytes with clear sarcomere striations was achieved. Only rod-shaped myocytes with clear edges were selected for contractile and intracellular Ca^2+^ studies^29^.

### Cell shortening/relengthening

Mechanical properties of cardiomyocytes were assessed using a SoftEdge MyoCam® system (IonOptix Corporation, Milton, MA, USA). IonOptix SoftEdge software was used to capture changes in cardiomyocyte length during shortening and re-lengthening. In brief, cardiomyocytes were placed in a Warner chamber mounted on the stage of an inverted microscope (Olympus, IX-70, Tokyo, Japan) and superfused (1 ml/min at 25 °C) with a buffer containing (in mM): 131 NaCl, 4 KCl, 1 CaCl_2_, 1 MgCl_2_, 10 glucose, 10 HEPES, at pH 7.4. Cells were field stimulated with supra-threshold voltage at a frequency of 0.5 Hz (unless otherwise stated), 3 msec duration, using a pair of platinum wires placed on opposite sides of the chamber connected to a FHC stimulator (Brunswick, NE, USA). The myocyte being studied was displayed on the computer monitor using an IonOptix MyoCam camera. IonOptix SoftEdge software was used to capture changes in cell length during shortening and relengthening. Cell shortening and relengthening were assessed using the following indices: peak shortening (PS) – the amplitude myocytes shortened on electrical stimulation, which is indicative of peak ventricular contractility; time-to-PS (TPS) – duration of myocyte shortening, which is indicative of contraction duration; time-to-90% relengthening (TR_90_) – duration to reach 90% relengthening, which represents cardiomyocyte relaxation duration (90% rather 100% relengthening was used to avoid noisy signal at baseline concentration); and maximal velocities of shortening (+dL/dt) and relengthening (−dL/dt) – maximal slope (derivative) of shortening and relengthening phases, which are indicatives of maximal velocities of ventricular pressure rise/fall^14^.

### Intracellular Ca^2+^ transient measurement

Cardiomyocytes were loaded with fura-2/AM (0.5 μM) for 10 min and fluorescence measurements were recorded with a dual-excitation fluorescence photomultiplier system (IonOptix). Cardiomyocytes were placed on an Olympus IX-70 inverted microscope and imaged through a Fluor ×40 oil objective. Cells were exposed to light emitted by a 75 W lamp and passed through either a 360 or a 380 nm filter, while being stimulated to contract at 0.5 Hz. Fluorescence emissions were detected between 480 and 520 nm by a photomultiplier tube after first illuminating the cells at 360 nm for 0.5 sec then at 380 nm for the duration of the recording protocol (333 Hz sampling rate). The 360 nm excitation scan was repeated at the end of the protocol and qualitative changes in intracellular Ca^2+^ concentration were inferred from the ratio of fura-2 fluorescence intensity (FFI) at two wavelengths (360/380). Fluorescence decay time was measured as an indication of the intracellular Ca^2+^ clearing rate. Both single and bi-exponential curve fit programs were applied to calculate the intracellular Ca^2+^ decay constant^14^.

### Histological examination

Histological analysis was performed as described^35^. Following anesthesia, hearts were arrested in diastole with saturated KCl, excised and fixed in 10% neutral-buffered formalin at room temperature for 24 hrs. The specimen was processed through graded alcohols, cleared in xylenes, embedded in paraffin, serial sections were cut at 5 μm and stained with FITC-tagged wheat germ agglutinin to examine cardiomyocyte size and Masson’s trichrome to evaluate interstitial fibrosis. Cardiomyocyte cross-sectional areas in cells with clear myofiber outlines and collagen volume fraction were measured on a digital microscope (×400) using the Image J (version1.34S) software.

### Western blot analysis

Murine heart tissues were homogenized and sonicated in RIPA buffer containing 20 mM Tris (pH 7.4), 150 mM NaCl, 1 mM EDTA, 1 mM EGTA, 1% Triton, 0.1% sodium dodecyl sulfate (SDS), and a protease inhibitor cocktail (Roche Diagnostics, Indianapolis, IN, USA). Heart homogenates containing equal amount of proteins were resolved by SDS-polyacrylamide gels in a mini-gel apparatus (Mini-PROTEAN II, Bio-Rad, Hercules, CA, USA) and the proteins were transferred to nitrocellulose membranes, incubated overnight with primary antibody at 4 °C. After being washed 3 times, the membrane was incubated with horseradish peroxidase (HRP)-coupled secondary antibody for 1 hr at room temperature. The membrane was washed again for 3 times 10 min each time, and the signal was detected quantified with a Bio-Rad Calibrated Densitometer and the intensity of immunoblot bands was normalized to that of GAPDH. For reprobing, membranes were tripped with 50 mmol/L Tris-HCl, 2% SDS and 0.1 M β-mercaptoethanol (pH 6.8). Polyclonal rabbit antibodies against Bcl-2, Bax, phosphorylated AMPK (pAMPKα) at Thr^172^, total AMPKα, phosphorylated mTOR (pmTOR) at Ser2448, total mTOR, LC3B, phosphorylated IĸBα at Ser^32/36^, IĸBα, TNFα, IL-1β, IL-6, IL-18, GAPDH and α-tubulin (1: 1,000; Rabbit; Cell Signaling Technology, Danvers, MA, USA); p62 (1: 1,000; Guinea Pig; Enzo Life Sciences, Plymouth Meeting, PA, USA); p16 and p21 (1:1,000; Rabbit; Proteintech Group, Chicago, IL, USA) were examined by standard immunoblotting. Membranes were probed respective antibodies with GAPDH or α-tubulin serving as the loading control^35^.

### Myocardial ATP level assay

Frozen heart tissue was homogenized in chloroform-methanol (1:20, w:v). The chloroform-rich layer was mixed with methanol following 15 min centrifugation at 15,000 g. The fluorescence in the sample was measured at an excitation wavelength of 350 nm and emission wavelength of 485 nm using a spectrofluorimeter (Molecular Devices, Sunnyvale, CA, USA)^6^,^22^. Data were expressed as fluorescence intensity per 100 mg tissue.

### Data analysis

Data were expressed as Mean ± SEM. The log rank test was used for Kaplan-Meier survival comparison. Statistical significance (p < 0.05) was estimated by one-way analysis of variation (ANOVA) followed by a Tukey’s test for *post hoc* analysis. All statistics was performed with GraphPad Prism 4.0 software (GraphPad, San Diego, CA, USA).

## Results

### MIF deficiency accentuates aging-induced changes in glucose handling capacity, myocardial structure and function

**Figure 1:**
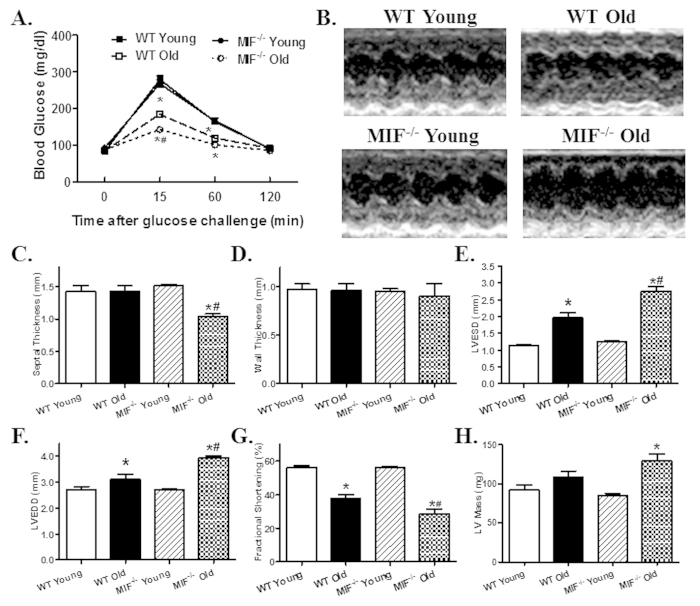
Intraperitoneal glucose tolerance test (IPGTT) and echocardiographic properties in young or old WT and MIF^−/−^ mice. (**A**) IPGTT (2 g/kg glucose challenge, i.p.) curve depicting glucose clearance capacity; (**B**) Representative M-mode echocardiographic images in hearts from young or old WT and MIF^−/−^ mice; (**C**) Septal thickness; (**D**) LV Wall thickness; (**E**) LV end systolic diameter (LVESD); (**F**) LV end diastolic diameter (LVEDD); (**G**) Fractional shortening; and H: LV mass. Mean ± SEM, n = 6 (panel **A**), 7–9 mice (Panel **C**–**H**) per group, *p < 0.05 *vs.* WT young group, ^#^p < 0.05 *vs.* WT old group.

**Figure 2:**
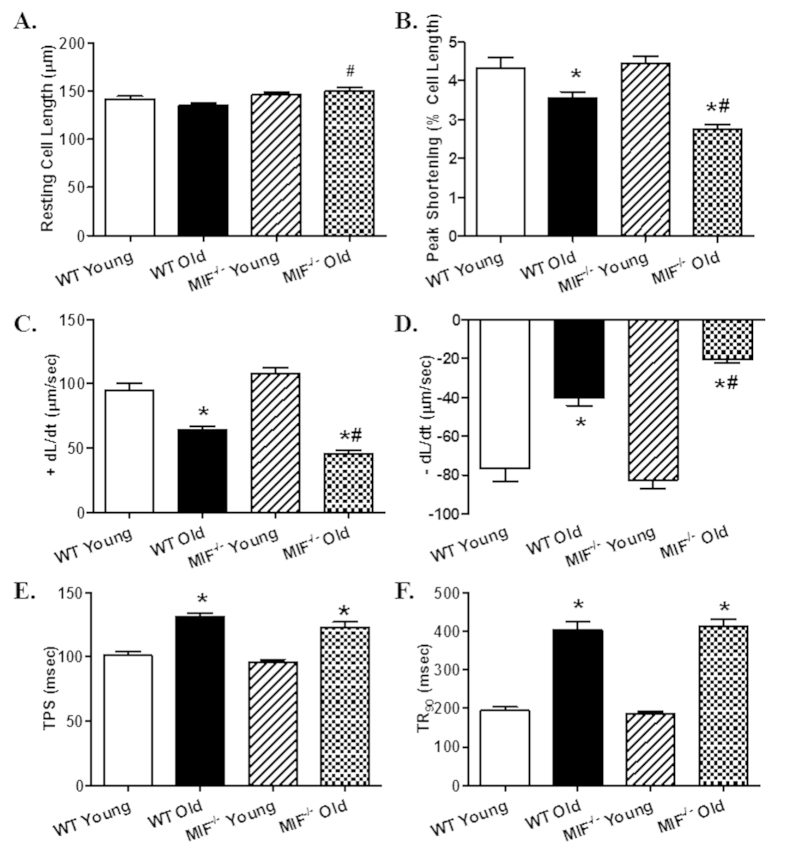
Cardiomyocyte contractile properties in young or old WT and MIF^−/−^ mice. (**A**) Resting cell length; (**B**) Peak shortening (PS, normalized to resting cell length); (**C**) Maximal velocity of cell shortening (+dL/dt); (**D**) Maximal velocity of cell relengthening (−dL/dt); (**E**) Time-to-peak shortening (TPS); and (**F**) Time-to-90% relengthening (TR_90_). Mean ± SEM, n = 90–101 cells per group, *p < 0.05 *vs.* WT young group, ^#^p < 0.05 *vs.* WT old group.

**Figure 3:**
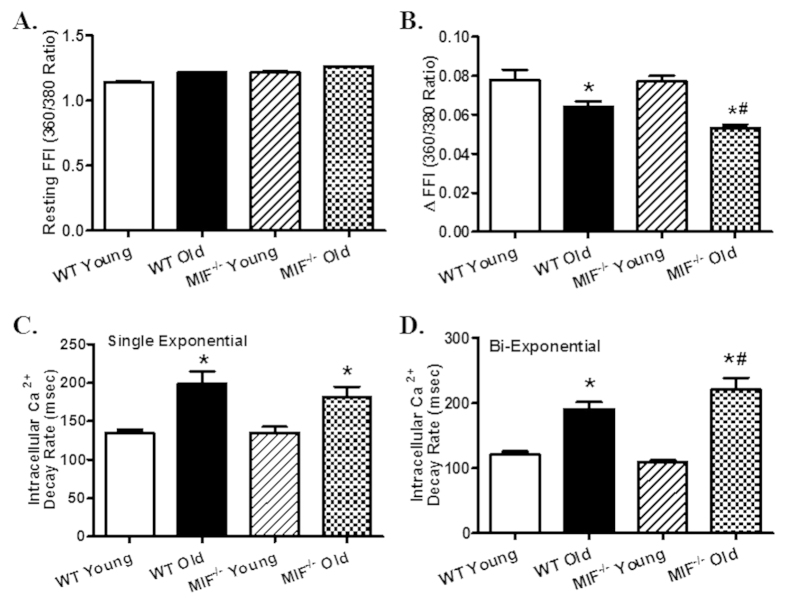
Intracellular Ca^2+^ handling properties in cardiomyocytes from young or old WT and MIF^−/−^ mice. (**A**) Resting Fura-2 fluorescence intensity (FFI); (**B**) Electrically-stimulated in FFI (ΔFFI); (**C**) Intracellular Ca^2+^ decay rate (single exponential); (**D**) Intracellular Ca^2+^ decay rate (bi exponential). Mean ± SEM, n = 89–119 cells per group, *p < 0.05 *vs.* WT young group, ^#^p < 0.05 *vs.* WT old group.

To assess glucose handling status, IPGTT was performed. While there was no difference in baseline blood glucose levels, blood glucose levels were overtly lower at 15 and 60 min following glucose challenge in aged mice compared with young ones with a more pronounced drop in aged MIF^−/−^ mice. MIF knockout itself did not affect the glucose handling capacity (Fig. 1A). Reminiscent of previous reports^5^,^6^, echocardiographic evaluation revealed significantly increased left ventricular (LV) end systolic diameter (ESD) and end diastolic diameter (EDD), as well as decreased fractional shortening associated with unchanged LV wall thickness, septal thickness and LV mass in old WT mice. Although MIF knockout itself did not overtly affect cardiac geometry in young mice, it exacerbated aging-induced unfavorable changes in cardiac geometry and function with more pronounced or unmasked changes in septal thickness, LVEDD, LVESD, fractional shortening and LV mass (Fig. 1B–H). Consistent with the echocardiographic findings, aging markedly suppressed cardiomyocyte contractile function, as evidenced by decreased peaking shortening (PS) and maximal velocity of shortening/relengthening (±dL/dt), as well as prolonged TPS and TR_90_. MIF deficiency overtly accentuated aging-induced cardiomyocyte contractile anomalies (more pronounced depression in PS and ±dL/dt) without elicit any effect itself in young mice. Resting cell length was not affected by either aging or MIF deficiency (Fig. 2A–F). To explore possible the mechanisms behind MIF deficiency-accentuated cardiac aging, intracellular Ca^2+^ handling was evaluated using Fura-2 fluorescence technique. Our data revealed that neither aging nor MIF deficiency, or both, significantly affected resting intracellular Ca^2+^ levels. Aging overtly decreased electrically-stimulated rise in intracellular Ca^2+^ (ΔFFI) and intracellular Ca^2+^ clearance rate (either single or bi-exponential) with a more pronounced change in MIF^−/−^ mice with the exception of single-exponential intracellular Ca^2+^ clearance. MIF deficiency itself did not affect the intracellular Ca^2+^ handling properties (Fig. 3A–D).

### Effect of MIF deficiency on aging-induced changes in cardiac morphology and biometrics

**Figure 4:**
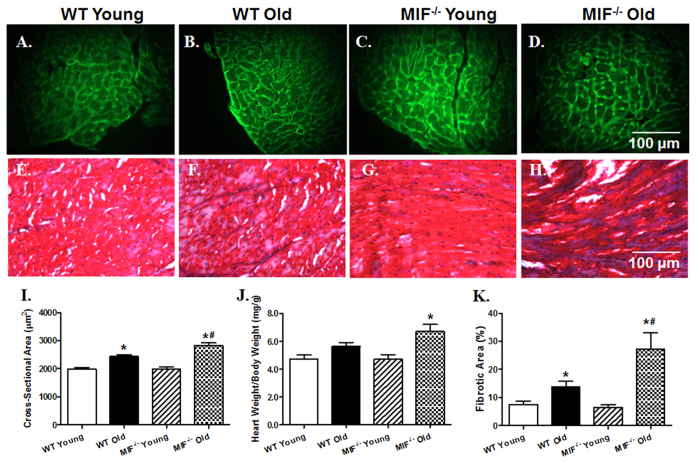
Myocardial morphology, biometrics and interstitial fibrosis in hearts from young or old WT and MIF^−/−^ mice. (**A**–**D**) Representative FITC-conjugated Lectin immunostaining depicting transverse sections of left ventricular myocardium (×400) in young or old WT and MIF^−/−^ mice; (**E**–**H**) Representative Masson Trichrome staining depicting interstitial fibrosis of myocardium (×400) in young or old WT and MIF^−/−^ mice; (**I**) Quantitative analysis of cardiomyocyte cross-sectional area; (**J**) Heart weight-to-body weight ratio; and (**K**) Quantitative analysis of interstitial fibrosis. Mean ± SEM, n = 6 mice per group for panels I and K, 11–12 mice per group for panel (**J**), *p < 0.05 *vs.* WT young group, ^#^p < 0.05 *vs.* WT old group.

Assessment of cardiomyocyte cross-sectional area using Lectin immunostaining revealed that aging significantly elevated cardiomyocyte cross-sectional area with a more pronounced rise in MIF^−/−^ mice. Likewise, aging exhibited a trend of increased heart-to-body weight ratio, with a more pronounced (and significant rise) in MIF^−/−^ mice. In addition, aging promoted myocardial interstitial fibrosis measured by Masson Trichrome staining with a more pronounced effect in MIF^−/−^ mice. MIF deficiency itself did not affect cardiomyocyte cross-sectional area, heart-to-body weight ratio or myocardial interstitial fibrosis in young mice (Fig. 4).

### Effect of MIF deficiency on cardiac senescence and myocardial autophagy

**Figure 5:**
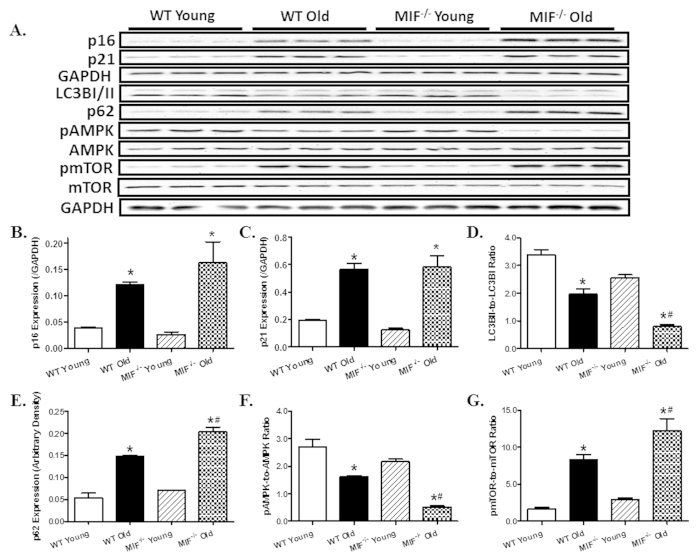
Effect of MIF deficiency on aging-induced changes in senescence marker, autophagy and autophagy regulatory molecules. (**A**) Representative gel blots depicting levels of the senescence markers p16 and p21, the autophagy markers LC3B and p62; pan and phosphorylated AMPK and mTOR using specific antibodies (GAPDH was used as loading control); (**B**) p16 expression; (**C**) p21 expression; (**D**) LC3BI/II expression (LC3BII-to-LC3BI ratio); (**E**) p62 expression; (**F**) pAMPK-to-AMPK ratio; and (**G**) pmTOR-to-mTOR ratio. Mean ± SEM, n = 3–4 mice per group, *p < 0.05 *vs.* WT young group, ^#^p < 0.05 *vs.* WT old group.

We examined levels of CDK (cyclin-dependent kinase) inhibitors including p16 and p21, both of which are used as the marker of cellular senescence. Our result showed that the protein levels of p16 and p21 were dramatically elevated with aging, the effect of which was unaffected by MIF deficiency. MIF deficiency itself did not affect myocardial p16 and p21 protein levels in young mice. Given that MIF is known to regulate autophagy^18^,^29^, which plays an important role in cardiac homeostasis maintenance^11^, myocardial autophagy was evaluated in hearts from young and aged WT and MIF^−/−^ mice. Our result showed decreased cardiac autophagy with aging as evidenced by decreased LC3BII-to-LC3BI ratio along with increased accumulation of p62 (an autophagy adaptor protein specifically degraded by autophagolysosome), the effect of which was exacerbated by MIF deficiency. Aging decreased AMPK phosphorylation and promoted mTOR phosphorylation without affecting pan protein expression of AMPK and mTOR. Although MIF deficiency itself did not alter phosphorylation of AMPK and mTOR, it significantly augmented aging-induced suppression of AMPK and elevation of mTOR (Fig. 5).

### MIF regulates doxorubicin-induced premature senescence in H9C2 cells

**Figure 6:**
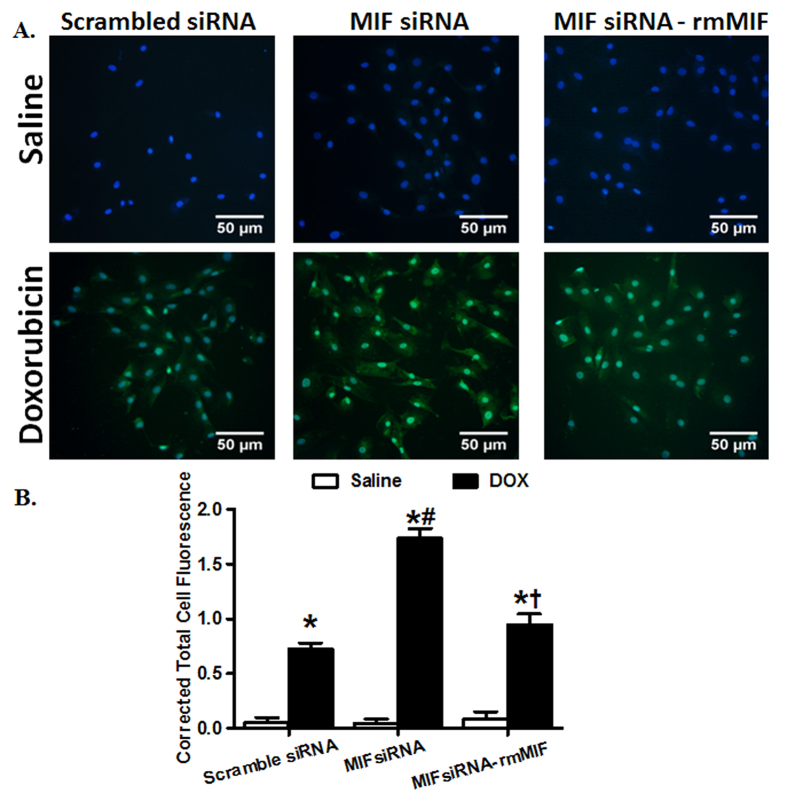
Effect of MIF silencing and replenishment on doxorubicin-induced cell senescence in cultured H9C2 myoblast cells. (**A**) Representative micrographs depicting levels of S-A β-gal measured using C_12_FDG (33 μM) in H9C2 cells. H9C2 cells treated with MIF siRNA or scrambled siRNA for 48 hrs were incubated with doxorubicin (DOX, 0.1 μM) for 24 hrs, followed by standard (DMEM without FBS) culture media for another 24 hrs. Recombinant mouse MIF (rmMIF, 10 ng/ml) was added into H9C2 cells treated with scrambled or siRNA MIF; and (**B**) Quantitative analysis of S-A β-gal levels in H9C2 cells. Mean ± SEM, n = 50 cells per group, *p < 0.05 *vs.* control group, ^#^p < 0.05 *vs.* DOX-scramble siRNA group, ^†^p < 0.05 *vs.* DOX MIF siRNA group.

To elucidate the potential effect of MIF in regulating cardiac aging, H9C2 myoblast cells were treated with doxorubicin (0.1 μM, 24 hrs) to induce premature senescence^31^,^32^,^33^. In addition, the endogenous MIF was depleted using MIF siRNA in H9C2 cells^18^. C_12_FDG, a fluorogenic substrate for β-galactosidase, was used to detect SA-β-gal as a marker for cellular senescence^22^. As expected, doxorubicin treatment induced premature senescence in H9C2 cells, as evidenced by accumulation of SA-β-gal, consistent with the well-established model of doxorubicin-induced cell senescence^31^,^33^. Although MIF depletion using siRNA did not affect cellular senescence by itself, it markedly exacerbated doxorubicin-induced cell senescence in H9C2 cells. To further consolidate the indispensable role of MIF in the process of senescence, H9C2 cells depleted of MIF were treated with recombinant mouse MIF (rmMIF, 50 ng/ml) prior to the challenge of doxorubicin. While rmMIF itself failed to elicit any effect in H9C2 cells with or without doxorubicin treatment, it abrogated MIF depletion-exacerbated detrimental effect of doxorubicin in H9C2 cells, as evidenced by attenuating the accumulation of SA-β-gal (Fig. 6).

### Rapamycin protects against the detrimental effects of MIF deficiency on cardiac aging

**Figure 7:**
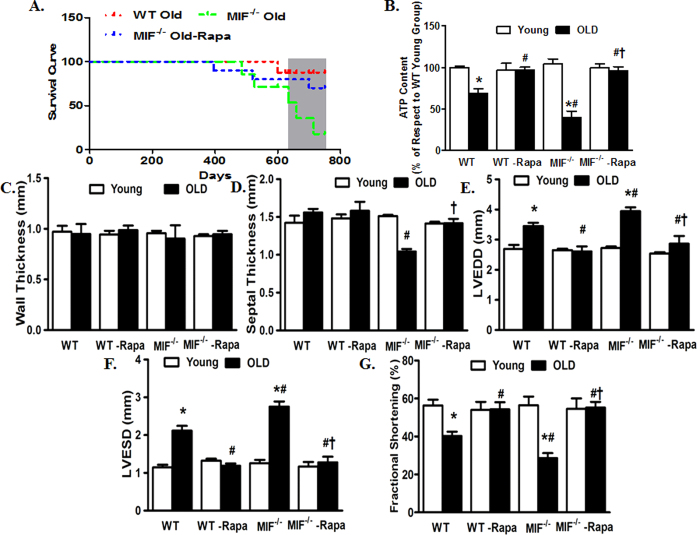
Kaplan Meier survival, myocardial ATP levels and echocardiographic properties in young or old WT and MIF^−/−^ mice treated with or without the autophagy inducer rapamycin. Rapamycin treatment (2 mg/kg body weight/d, i.p.) were initiated when MIF^−/−^ mice were 22-month old for 2-month. (**A**) Kaplan-Meier survival curve of old WT and MIF^−/−^ mice as well as old MIF^−/−^ mice treated with rapamycin (Rapa); (**B**) Myocardial ATP levels; (**C**) LV Wall thickness; (**D**) Septal thickness; (**E**) LV end diastolic diameter (LVEDD); (**F**) LV end systolic diameter (LVESD); and (**G**) Fractional shortening. Mean ± SEM, n = 7–9 mice per group, *p < 0.05 *vs.* WT young group, ^#^p < 0.05 *vs.* WT old group, ^†^p < 0.05 *vs.* MIF^−/−^ old group.

**Figure 8:**
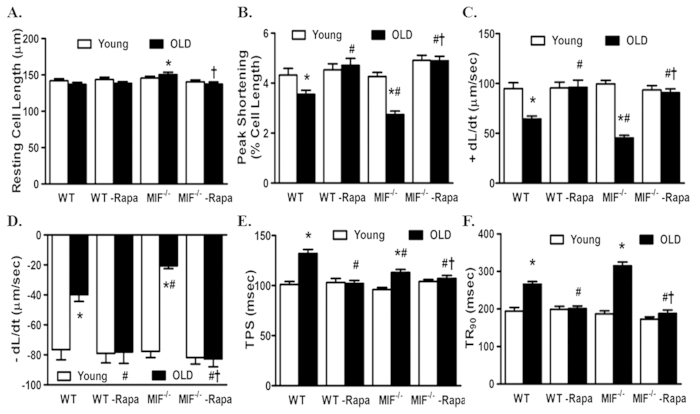
Cardiomyocyte contractile properties in young or old WT and MIF^−/−^ mice treated with or without the autophagy inducer rapamycin. Rapamycin treatment (2 mg/kg body weight/d, i.p.) were initiated when MIF^−/−^ mice were 22-month old for 2-month. (**A**) Resting cell length; (**B**) Peak shortening (PS, normalized to resting cell length); (**C**) Maximal velocity of shortening (+dL/dt); (**D**) Maximal velocity of relengthening (−dL/dt); (**E**) Time-to-peak shortening (TPS); and (**F**) Time-to-90% relengthening (TR_90_). Mean ± SEM, n = 90–107 cells per group, *p < 0.05 *vs.* WT young group, ^#^p < 0.05 *vs.* WT old group, ^†^p < 0.05 *vs.* MIF^−/−^ old group.

Given that activated mTOR and suppressed autophagy were associated with exacerbated cardiac aging in MIF^−/−^ mice, we examined the effect of rapamycin, an inhibitor of mTOR and inducer of autophagy, on cardiac aging in WT and MIF^−/−^ mice. Young (3-month-old) or aged (22-month-old) WT and MIF^−/−^ mice were treated with rapamycin (2 mg/kg body weight/day, i.p.) for 8 weeks. Treatment with rapamycin dramatically increased survival of aged WT and MIF^−/−^ mice (Fig. 7A). To elucidate the beneficial effect of rapamycin-induced autophagy in rescuing cardiac function in aged WT and MIF^−/−^ mice, we evaluated myocardial ATP levels. Our data revealed that myocardial ATP level was dramatically decreased by aging, the effect of which was further accentuated by MIF knockout. Interestingly, rapamycin treatment significantly improved ATP levels in hearts from aged WT and MIF^−/−^ mice, although little effects were noted for rapamycin on myocardial ATP levels in young mice (Fig. 7B). We went on to evaluate the cardioprotective effect of rapamycin in aged WT and MIF^−/−^ mice. Our echocardiography data indicated that rapamycin abrogated aging-induced unfavorable cardiac geometric and functional changes including reduced fractional shortening and enlarged LV chambers (LVESD and LVEDD) in WT and MIF^−/−^ mice (Fig. 7C–G). Moreover, cardiomyocyte mechanical properties from young and aged WT and MIF^−/−^ mice with or without rapamycin treatment were evaluated. Consistently, rapamycin protected against aging-induced cardiomyocyte contractile dysfunction such as reduced PS and ±dL/dt and prolonged TR_90_ in aged WT and MIF^−/−^ mice. Aging and MIF deficiency did not overtly affect the resting cell length although the combination of the two significantly prolonged the cell length in cardiomyocytes, the effect of which was abolished by rapamycin treatment (Fig. 8A–F). Rapamycin itself did not elicit any notable effect on cardiac geometry and contractile function as well as cardiomyocyte mechanical function in young WT and MIF^−/−^ mice (Figs 7 and 8).

### Effect of MIF deficiency aging-induced cardiac inflammation

**Figure 9:**
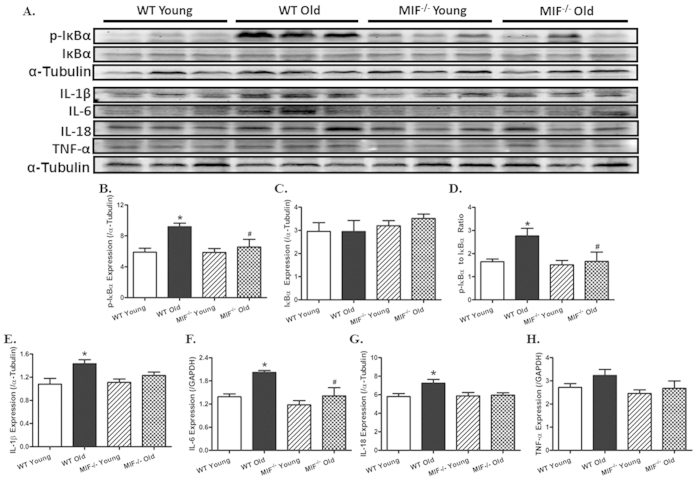
Effect of MIF deficiency on aging-induced changes in proinflammatory markers in hearts including IκB, phosphorylated IκB (p-IκB), IL-1β, IL-6, IL-18 and TNF-α. (**A**) Representative gel blots depicting levels of IκB, p-IκB, IL-1β, IL-6, IL-18 and TNF-α using specific antibodies (α-tubulin was used as the loading control); (**B**) p-IκB expression; (**C**) IκB expression; (**D**) p-IκB-to-IκB ratio; (**E**) IL-1β expression; (**F**) IL-6 expression; (**G**) IL-18 expression; and (**H**) TNF-α expression. Mean ± SEM, n = 5–6 mice per group, *p < 0.05 *vs.* WT young group, ^#^p < 0.05 *vs.* WT old group.

Given that MIF is a pro-inflammatory cytokine^22^,^23^,^28^, myocardial inflammation was examined on young and aged mice with or without MIF knockout. Our result showed that aging significantly promoted phosphorylation of the NFκB inhibitor IκB (absolute or normalized to pan IκB level), denoting for a higher pro-inflammatory NFκB phosphorylation (activity) state. MIF deficiency itself did not affect IκB phosphorylation in young mice, although it abrogated aging-induced IκB phosphorylation. Neither aging nor MIF deficiency significantly affected the pan IκB expression (Fig. 9A–D). Along the same line, advanced aging significantly upregulated the myocardial proinflammatory protein makers IL-1β, IL-6 and IL-8 (with a non-significant trend of elevated TNF-α levels), the effects of which were ablated or significantly attenuated by MIF knockout. MIF deficiency itself did not exert any notable effect on these proinflammatory protein makers in the heart in young mice (Fig. 8A,E–H).

### Rapamycin failed to affect MIF deficiency-induced inflammatory responses in cardiac aging

**Figure 10:**
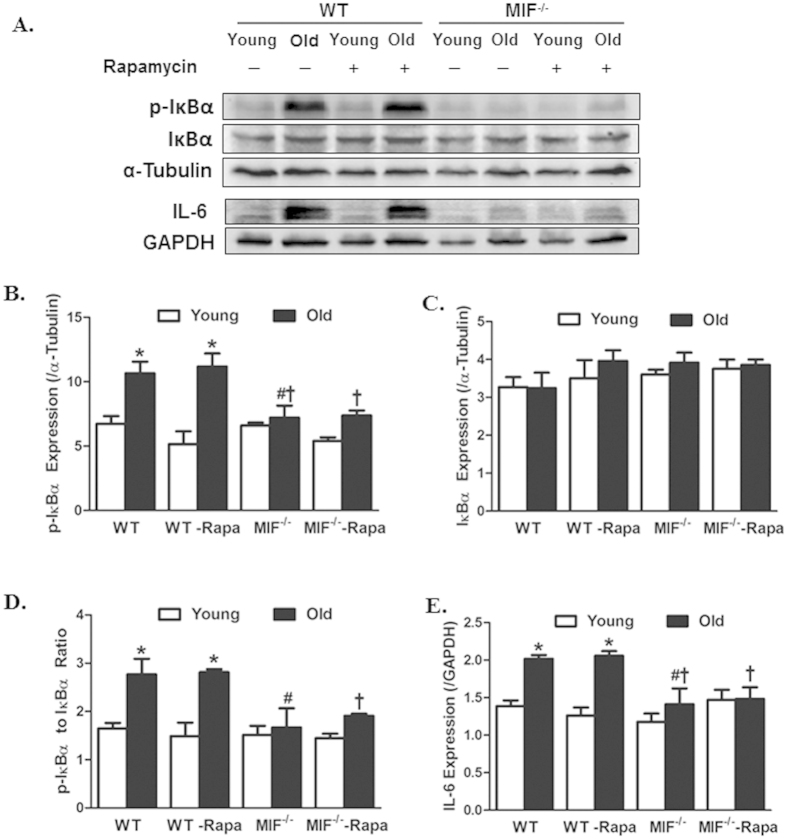
Levels of proinflammatory protein markers in hearts from young or old WT and MIF^−/−^ mice treated with or without the autophagy inducer rapamycin. Rapamycin treatment (2 mg/kg body weight/d, i.p.) were initiated when MIF^−/−^ mice were 22-month old for 2-month. (**A**) Representative gel blots depicting levels of IκB, p-IκB and IL-6 using specific antibodies (α-tubulin and GAPDH were used as the loading control); (**B**) p-IκB expression; (**C**) IκB expression; (**D**) p-IκB-to-IκB ratio; and (**D**) IL-6 expression; Mean ± SEM, n = 3–6 mice per group, *p < 0.05 *vs.* WT young group, ^#^p < 0.05 *vs.* WT old group, ^†^p < 0.05 *vs.* MIF^−/−^ old group.

To examine if changes in cardiac inflammation play any role in the autophagy induction-induced beneficial effect against aging, young (3-month-old) or aged (22-month-old) WT and MIF^−/−^ mice were treated with rapamycin (2 mg/kg body weight/day, i.p.) for 8 weeks prior to assessment of proinflammatory markers. Our results revealed that rapamycin failed to affect MIF deficiency-offered beneficial effect against aging-induced inflammation (as manifested by IκB phosphorylation (absolute or normalized to pan IκB) and IL-6 expression. Neither aging nor MIF deficiency affected the levels of pan IκB, the response pattern was unaffected by rapamycin. Rapamycin treatment by itself did not elicit any response on cardiac pro-inflammatory protein markers (Fig. 10A–E). These findings suggest that rapamycin-offered beneficial response against cardiac aging in both WT and MIF^−/−^ mice is unlikely mediated through cardiac inflammation.

## Discussion

The salient findings from our study indicated that MIF deficiency accentuated aging-induced cardiac remodeling, contractile dysfunction, and intracellular Ca^2+^ mishandling despite improved myocardial inflammation. MIF deficiency overtly worsened the aging-induced loss of autophagy, including buildup of insoluble p62, favoring a role for autophagy regulation in MIF deficiency-accentuated cardiac aging. Along the same line, aging-induced and MIF-accentuated unfavorable functional responses (and survival) were reversed by autophagy induction using the mTOR inhibitor rapamycin. However, autophagy induction failed to affect MIF knock-induced cardiac inflammation response in aging, suggesting the rapamycin- and MIF knockout-induced mechanical responses were likely independent of inflammation regulation. The involvement of autophagy in MIF knockout-accentuated cardiac aging was further substantiated by *in vitro* findings where MIF knockdown aggravated while recombinant MIF replenishment alleviated cell senescence in an established doxorubicin-induced premature cell senescence model^31^,^32^,^33^. These results collectively favored a role for the endogenous proinflammatory cytokine MIF in the homeostasis of autophagy, myocardial morphology and function, independent of inflammation regulation during the aging process and more importantly, the therapeutic potential targeting MIF in cardiac aging.

Unfavorable morphological and functional changes have been documented in aged hearts characterized by cardiac remodeling (hypertrophy and interstitial fibrosis), intracellular Ca^2+^ mishandling, compromised myocardial contractility and prolonged systolic/diastolic duration^36^,^37^,^38^,^39^. Compromised myocardial contractile function and geometry are seen in senescence, leading to defects in early diastolic filling and overall pump function^38^,^40^,^41^,^42^. In our hands, overt cardiac remodeling (enlarged LVESD, LVEDD) and pump dysfunction (decreased fractional shortening) were noted in aged hearts, the effects of which were aggravated by MIF deficiency. Although aging failed to alter LV wall thickness, septal thickness and LV mass, MIF deficiency unmasked aging-associated a drop in septal thickness and an elevation in LV mass (supported by the FITC-tagged wheat germ agglutinin staining and heart-to-body weight ratio). These MIF deficiency-offered changes in cardiac structure and function in advanced aging are somewhat reminiscent of the exacerbated myocardial responses under pressure overload^29^. These MIF deficiency-induced prominent echocardiographic changes are also supported by the more pronounced myocardial interstitial fibrosis in aged MIF^−/−^ mice, favoring a “permissive” role for MIF in aging as opposed to the young ones. Ventricular hypertrophy and interstitial fibrosis are hallmarks for cardiac aging, contributing to the onset and progression of heart failure^43^. Our data revealed that aged MIF^−/−^ hearts possess greater heart mass, heart-to-body weight ratio, cardiomyocyte size and interstitial fibrosis compared with aged WT mice. Although it is beyond the scope of our study to examine the precise mechanism responsible for cardiac hypertrophy in aged MIF^−/−^ mice, deletion of the pro-inflammatory cytokine MIF in the heart may have break the balance between pro- and anti-hypertrophic cytokines in the heart with advanced aging. This is supported by inflammatory protein markers where MIF deficiency disrupted the inflammatory status in aged but not young hearts. Earlier finding from our group revealed that aging process overtly decreased cardiac MIF levels^30^, suggesting a possible “double whammy” for MIF homeostasis in aged MIF knockout mice. It is worth mentioning that young MIF^−/−^ mice exhibited comparable body and heart weights, glucose handling capacity and cardiac ATP levels compared with their WT littermates, suggesting that deficiency of this pro-inflammatory cytokine early on in life may not be innately harmful for cardiac homeostasis. Interestingly, IPGTT curve revealed that aged MIF^−/−^ mice displayed a subtle although more pronounced drop in glucose handling compared with their age-matched WT mice, denoting a possible role for MIF in glucose metabolism. MIF has been demonstrated to regulate glucose metabolism through a number of mechanism including activation of AMPK^28^,^30^ and facilitation of GLUT4 glucose transport^44^ in cardiomyocytes. Thus a possible cardiac effect secondary to changes in global glucose metabolism cannot be ruled out for MIF knockout at this time.

Earlier findings from our laboratory suggested that endogenous MIF protects against pathological myocardial changes in pressure overload where it perturbs^15^ cardiac dysfunction in high fat diet-induced obesity, mainly through regulation of autophagy^29^. Here advanced aging-induced cardiac anomalies were accentuated by MIF deficiency, as evidenced by exacerbated myocardial geometric, functional and histological changes. Paradoxically, MIF knockout overtly lessened aging-induced cardiac inflammation as evidenced by NFκB phosphorylation (using IκB phosphorylation as an indicator) and the pro-inflammatory cytokines IL-1β, IL-6, IL-6, IL-18 and TNF-α. Aging is associated with profound inflammation in the heart delaying the resolving response after myocardial infarction^45^. It is not surprising that deficiency of the proinflammatory cytokine MIF alleviates inflammation in aged hearts. Our data showed dampened autophagy in aged hearts. Maintaining autophagy is pivotal in the maintenance of cardiac homeostasis under various stress conditions including ischemia/reperfusion^46^, hypertrophy^13^ and pressure overload-induced heart failure^47^. Our findings demonstrated that aging dramatically dampened cardiac autophagy, the effect of which was significantly accentuated by MIF knockout. Assessment of autophagy regulatory pathways revealed that aging triggered deactivation of the AMPK-mTOR-autophagy pathway, the effect of which was augmented by MIF deficiency. Interestingly, administration of rapamycin, an mTOR inhibitor and autophagy inducer, reversed the cardiac remodeling and contractile dysfunction without affecting cardiac inflammation status in aged hearts, consistent with the findings that rapamycin reverses established cardiac remodeling and contractile dysfunction^48^,^49^,^50^, in particular in pressure overload pathology under MIF deficiency^29^. Our finding also suggested that treatment with the autophagy inducer rapamycin significantly improved mouse survival and cardiac contractile function along with myocardial ATP levels in aged WT and MIF^−/−^ mice, suggesting a possible role for ATP as a downstream effector in MIF deficiency-exacerbated and rapamycin-protected cardiac contractile response. Autophagy is well known to regenerate ATP for cardiac homeostasis^10^,^11^. AMPK serves as an important inducer of autophagy, while mTOR is a primary inhibitor of autophagy. Activation of AMPK retards cardiac aging in animal models^9^,^30^,^51^,^52^. Among various downstream regulators of AMPK, mTOR plays an important role in aging-induced cardiac remodeling and inhibition of mTOR is believed to be responsible for the anti-aging effect of AMPK activators such as resveratrol^6^,^53^,^54^. MIF is an important mediator of AMPK activation^28^,^29^,^30^,^55^. Our data strongly argue that MIF regulates the AMPK-mTOR-autophagy pathway in cardiac remodeling and dysfunction in aging. Upregulation of autophagy may be beneficial for the management of cardiac remodeling and heart dysfunction, in particularly in those individuals who are genetic low producers of MIF^28^.

Our *in vitro* data using the established cell senescence model triggered by doxorubicin^31^,^32^,^33^ revealed that MIF knockdown and MIF replenishment exacerbated and alleviated, respectively, doxorubicin-induced premature cell senescence as measured using accumulation of S-A β-gal. Doxorubicin triggers a senescent-like phenotype in cardiomyocytes, with a disturbed pattern of troponin phosphorylation, resulting in inefficient cardiac contraction^31^,^33^. Primary neonatal cardiomyocytes display markers of senescence and reduced telomerase activity with incubation of doxorubicin^31^. In addition, doxorubicin promotes senescence of resident cardiac progenitor cells (CPCs) which are supposed to preserve myocardial homeostasis following stress^56^. These data supported the *in vivo* data in that premature cardiac aging (cell senescence) may be exacerbated by MIF deficiency. MIF deficiency-induced cell senescence may be reversed by recombinant MIF replenishment in H9C2 myoblast cells. Future work is warranted to examine how MIF regulates biological aging process.

**Figure 11:**
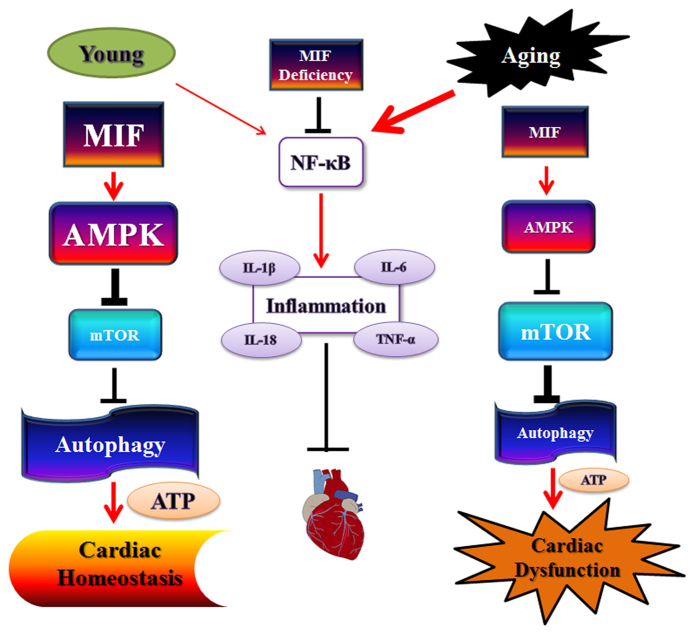
Schematic diagram depicting the proposed differential regulations of MIF-induced of control of the AMPK-mTOR-dependent autophagy pathways in cardiac homeostasis (young) and cardiac dysfunction (aging). Aging suppresses MIF levels, leading to a less pronounced AMPK activation. Activation of AMPK inhibits mTOR and turns on autophagy, thus resulting in the preserved ATP content and cardiac homeostasis. AMPK-mediated autophagy and ATP content are reduced in aging, prompting cardiac dysfunction. Box, letter and line sizes refer to the levels or potencies of signaling molecules or events. Aging promotes IκB/NFκB-mediated myocardial inflammation, the effect of which is alleviated by MIF deficiency independent of autophagy process. Arrows denote stimulation whereas “T” ending represent inhibition. MIF: Microphage migration factor, AMPK: AMP-activation protein kinase; mTOR: mammalian target of rapamycin.

In summary, the findings from our current study reveal MIF deficiency exacerbates aging -induced cardiac remodeling and contractile dysfunction despite improved cardiac inflammatory status, possibly through suppression of autophagy (Fig. 11). This finding is indicative for an indispensable role for endogenous MIF in the homeostasis of cardiac geometry and function in aging in a manner reminiscent of ischemia/reperfusion and pressure overload^28^,^29^,^30^,^55^. Restoration of autophagy using rapamycin rescues cardiac geometric and functional sequelae without affecting cardiac inflammation status in aged WT and MIF^−/−^ mice. These findings suggest that elderly individuals with low levels or mutated *MIF* alleles may be prone to cardiac remodeling and contractile anomalies. In addition, our observations should shed some promises of autophagy induction as a potential therapeutic strategy for the management of cardiac remodeling and dysfunction in the elderly.

## Additional Information

**How to cite this article**: Xu, X. *et al.* Macrophage Migration Inhibitory Factor (MIF) Deficiency Exacerbates Aging-Induced Cardiac Remodeling and Dysfunction Despite Improved Inflammation: Role of Autophagy Regulation. *Sci. Rep.* **6**, 22488; doi: 10.1038/srep22488 (2016).
